# Molecular profiling of signalling proteins for effects induced by the anti-cancer compound GSAO with 400 antibodies

**DOI:** 10.1186/1471-2407-6-155

**Published:** 2006-06-09

**Authors:** Verity A Cadd, Philip J Hogg, Adrian L Harris, Stephan M Feller

**Affiliations:** 1 Cancer Research UK Cell Signalling Group, Weatherall Institute of Molecular Medicine, University of Oxford, UK; 2 Centre for Vascular Research, University of New South Wales, Sydney 2052 and Children's Cancer Institute Australia for Medical Research, Randwick 2031, Australia; 3 Cancer Research UK Growth Factor Group, Weatherall Institute of Molecular Medicine, University of Oxford, UK

## Abstract

**Background:**

GSAO (4-[N-[S-glutathionylacetyl]amino] phenylarsenoxide) is a hydrophilic derivative of the protein tyrosine phosphatase inhibitor phenylarsine oxide (PAO). It inhibits angiogenesis and tumour growth in mouse models and may be evaluated in a phase I clinical trial in the near future. Initial experiments have implicated GSAO in perturbing mitochondrial function. Other molecular effects of GSAO in human cells, for example on the phosphorylation of proteins, are still largely unknown.

**Methods:**

Peripheral white blood cells (PWBC) from healthy volunteers were isolated and used to profile effects of GSAO vs. a control compound, GSCA. Changes in site-specific phosphorylations, other protein modifications and expression levels of many signalling proteins were analysed using more than 400 different antibodies in Western blots.

**Results:**

PWBC were initially cultured in low serum conditions, with the aim to reduce basal protein phosphorylation and to increase detection sensitivity. Under these conditions pleiotropic intracellular signalling protein changes were induced by GSAO. Subsequently, PWBC were cultured in 100% donor serum to reflect more closely *in vivo *conditions. This eliminated detectable GSAO effects on most, but not all signalling proteins analysed. Activation of the MAP kinase Erk2 was still observed and the paxillin homologue Hic-5 still displayed a major shift in protein mobility upon GSAO-treatment. A GSAO induced change in Hic-5 mobility was also found in endothelial cells, which are thought to be the primary target of GSAO *in vivo*.

**Conclusion:**

Serum conditions greatly influence the molecular activity profile of GSAO *in vitro*. Low serum culture, which is typically used in experiments analysing protein phosphorylation, is not suitable to study GSAO activity in cells. The signalling proteins affected by GSAO under high serum conditions are candidate surrogate markers for GSAO bioactivity *in vivo *and can be analysed in future clinical trials. GSAO effects on Hic-5 in endothelial cells may point to a new intracellular GSAO target.

## Background

The term 'cancer' encompasses a wide variety of distinct, multigenic diseases. Even within a specific tumour type, a remarkable degree of heterogeneity on the level of DNA lesions and affected signalling pathways is apparent. Many cancer relevant signalling molecules, but also many molecular targets of anti-cancer drugs, therefore remain unknown. Prominent examples of signalling protein classes long known to be involved in generating cancer pathologies include GTPases, protein kinases and transcription factors. By contrast, protein phosphatases have only recently entered the stage as known players in cancer development. At least 30 protein phosphatases are now implicated in cancer development and other human diseases [1-3]. In some of these cases, mutational inactivation of a protein phosphatase appears to mimic the constitutive activation of its target kinase(s) [3]. In other cases, hyperactivation or deregulation of a phosphatase may contribute to kinase activation. For example, overexpression of the Cdc25 family phosphatases, which control cell cycle progression, is well documented for a variety of cancers, making the Cdc25 proteins interesting potential targets for anti-cancer therapies [4-7] and references therein).

The modulation of specific cellular signalling pathways to treat human cancers has only recently developed into an area of intense clinical research activity. A large number of clinical trials for novel signal transduction modulator (STM) drugs are currently planned or under way. STM drugs often have relatively low toxicity, so determination of the maximum tolerated dose (MTD) may not be a prime goal for phase I clinical trials. Instead, identification of an optimal biologically active dose (OBD) is essential [8]. Rapid determination of the OBD requires that *in vivo *markers of drug activity are available before or very early during the clinical trial.

**Figure 1:**
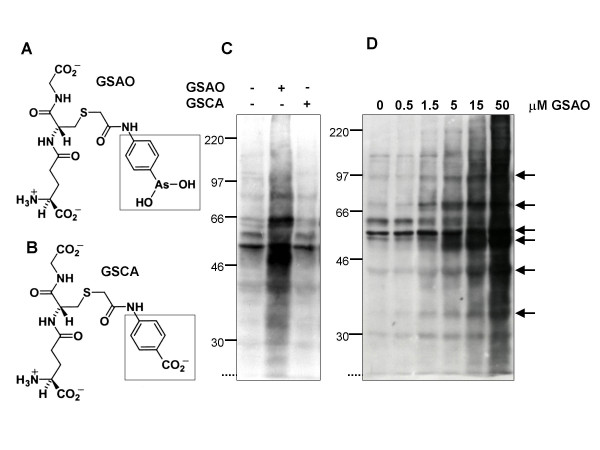
**Chemical structure GSAO and GSCA and Dose-dependent increase of PWBC protein tyrosine phosphorylation induced by GSAO**. **A **and **B**. The boxes indicate the region of the molecule thought to be primarily responsible for the biological activity of GSAO and the corresponding region in the control GSCA. Oxidation of GSAO is possible. The resulting molecule (GSAA) is thought to be inactive, but may become active again, if reduced *in vivo*. To avoid oxidation, GSAO solutions are prepared freshly, or stored after snap-freezing at -80°C. No significant activity loss of deep-frozen GSAO was observed for up to 6 months. PWBC were treated with GSAO or GSCA where indicated for 24 h and tyrosine phosphorylation detected by western blot. **C **50 μM GSCA does not alter the protein pTyr levels of PBWC detectably. **D **Changes in protein pTyr patterns are apparent with as little as 1.5 μM GSAO. GSAO-affected proteins are indicated with arrows.

This study identifies several proteins in PWBC which are affected by the novel anti-cancer compound GSAO (4-[N-[S-glutathionylacetyl]amino] phenylarsenoxide) [9] (Figure 1A). They may be useful as clinical surrogate markers to monitor or predict the anti-cancer activity of GSAO and could also help to provide further insight into the biological mechanisms of GSAO action.

GSAO has anti-angiogenic activity *in vitro *and *in vivo *[10]. Mitochondria and in particular the adenine nucleotide translocator (ANT) have been described as one target of GSAO. However, mitochondria are present in virtually all living cells. Therefore, inhibition of ANT does not *per se *explain the low toxicity and anti-angiogenic activity of GSAO.

Recent studies have revealed that differences in multidrug resistance proteins (MRP1 and 2) and cellular glutathione levels [11] appear to contribute to the preferential *in vivo *activity of GSAO in endothelial cells. Nevertheless, it is well known that practically all drugs in clinical use have multiple molecular *in vivo *targets. They depend on the affected cell type and applied doses. Some of these targets are intimately linked to the adverse effects of the drug. In cancer cells, with their heterogeneous genetic lesions, drug targets can even be present in different combinations or at different expression and activity levels when comparing individual patients. Identification and monitoring of novel molecular targets could thus help to understand why a drug is more effective or toxic in some patients compared to others. PWBC from donated blood were chosen for the analyses, since they are easily obtainable from healthy volunteers prior to clinical trial and later from the patients enrolled in the trials. A main focus of the experiments was to detect changes in protein phosphorylation induced by GSAO treatment, because GSAO is a hydrophilic derivative of phenylarsine oxide (PAO), a well known protein phosphatase inhibitor [12,13]. In contrast to GSAO, PAO is unsuitable for *in vivo *applications and toxic for cells at nanomolar concentrations [10]. It penetrates very rapidly into cell membranes and is hence not equally distributed throughout the vascular system of an organism. As GSAO is expected to enter a phase I clinical trial in the near future a molecular profiling of its effects on signalling proteins prior to the trial was performed. The aim was to provide data for trial-supporting pharmacodynamic molecular assays, trial design and possibly identification of additional mechanisms of GSAO action.

## Methods

### Isolation and culture of PWBC from healthy human volunteers

PWBC were obtained from 14 healthy volunteers not taking any medication (8 male, 6 female; aged between 23 and 60 years) with informed consent. Ethical approval was granted by the Oxfordshire Ethics Committee (study number 05/Q1605/59). Cell preparations from more than 40 different blood donations were used. All blood donations took place between 9 and 11 a.m. to minimise potential circadian changes of signalling proteins. Whole blood (54 ml) was collected from a peripheral arm vein into 6 ml of 46.7% (v/v) trisodium citrate (final concentration 4.67%) and syringes immediately placed on a nutator at room temperature for at least 5 min to ensure complete mixing. Batches of 20 ml citrated blood were then carefully layered onto 20 ml Polymorphprep™ density gradient media (Axis Shield PoC AS, Oslo, Norway) and centrifuged at 500 × g for 30 min at 20°C to remove erythrocytes. PWBC were collected from the medium/serum interface, transferred into a 50 ml centrifuge tube and gently resuspended in sterile PBS. PWBC were then centrifuged for 20 min at 600 × g at 20°C, PBS was aspirated and the PWBC pellet stored in ice. PWBC were subsequently cultured in either 100% donor serum or RPMI1640 supplemented with 0.5% foetal bovine serum (FBS), 100 μg/ml penicillin-streptomycin and 2 mM L-glutamate. Donor serum was simultaneously prepared freshly from peripheral blood. For this, blood was collected without anticoagulant and centrifuged for 30 min at 3700 × g at 20°C. The supernatant was transferred into a fresh centrifuge tube and incubated for 2 h at room temperature with nutation to allow coagulation, before centrifugation at 3700 × g for 30 min at 20°C to obtain serum. Pelleted PWBC were resuspended in either growth medium or donor serum and equilibrated for 2 h at 37°C in a humidified cell culture incubator with 5% CO_2_, before treatment with the compound of interest. Cells were treated with either GSAO, the active drug, or 4-N-(S-glutathionyl acetyl) amino) benzoic acid (GSCA) in which the trivalent arsenic of GSAO has been replaced by a carboxylic acid, as a control, for the concentrations and durations indicated. Stock solutions of both GSCA and GSAO were made at a concentration of 3 mM in RPMI 1640 and, since GSAO is prone to oxidation, stored in aliquots at -80°C unless used immediately. PAO was freshly dissolved in cell culture grade DMSO at a concentration of 10 mM and diluted to 1 μM with medium.

### Preparation and analyses of protein lysates

Treated or mock-incubated PWBC were scraped from tissue culture flasks, transferred to a 50 ml centrifuge tube filled with PBS and centrifuged at 3700 × g for 20 min at 20°C to pellet the cells. The PBS was completely aspirated from the PWBC pellet and the pellet lysed directly in boiling SDS-PAGE sample buffer (70 mM TrisHCl pH6.8, 5% (v/v) β-mercaptoethanol, 40% (v/v) glycerol, 3%(w/v) SDS, 0.05% (w/v) bromophenol blue). The samples were then boiled for a further 5 min, cooled to room temperature and sonicated for 30 seconds to fragment DNA contained in the cell lysate. Proteins in the lysate were resolved by SDS polyacrylamide gel electrophoresis (SDS-PAGE) and electrotransferred to PVDF membranes (Hybond P, Amersham Biosciences). The membranes were blocked and probed overnight at 4°C with different phospho-epitope specific or other antibodies according to the antibody manufacturers instructions. Bound primary antibodies were detected using horseradish peroxidase-conjugated anti-mouse or anti-rabbit immunoglobulins (Jackson ImmunoResearch Inc.) and ECL (Amersham Biosciences). Equal loading of different protein extract samples was routinely confirmed by Coomassie-blue staining of an SDS-PAGE-separated aliquot prior to blot analyses. Antibodies used in this study are compiled in the additional files 8 and 9. The BD PowerBlot Western Array Screen Service (BD Biosciences) was utilised to analyse expression levels of 240 individually selected signalling proteins using monoclonal antibodies.

### Culture and treatment of HUVEC

Human umbilical vein endothelial cells (HUVEC) were purchased from TCS Cell Works, (Botolph Claydon, UK) and cultured according to the manufacturers conditions with recommended growth media, cell attachment factor and passage conditions. Cells were cultured in 10 cm plates until 80% confluent before treatment with GSAO or GSCA in growth media with high serum (15% FBS) for 24 hours at 37°C with 5% CO_2_. Cells were lysed with boiling sample buffer. Protein analysis, SDS PAGE and Western blots were carried out as described for the PWBC.

## Results

### Pleiotropic effects of GSAO on PWBC cultured in low serum conditions

In initial experiments it was determined how rapidly protein phosphorylation changes were detectable upon GSAO treatment of freshly isolated PWBC from healthy donors under low serum culture conditions. For this, PWBC were incubated with GSAO or its more hydrophobic relative PAO. As previously reported in the literature for various cell types, increased tyrosine phosphorylation was rapidly observed with PAO. Incubation of PWBC with 1 μM PAO for 1 h resulted in a hyper-phosphorylation of protein-tyrosyl residues that was comparable to an incubation of PWBC with 50 μM GSAO for 8 h (additional file 1). This was not unexpected, since GSAO is specifically modified to greatly slow down its uptake into cells. Incubation of PWBC with a single dose of GSAO for 24 h instead of 8 h greatly enhanced the hyperphosphorylation of proteins on tyrosine residues (figure 1C). Treatment with 50 μM of the control compound GSCA (see figure 1B for structure), which lacks the crucial arsenic moiety, did not detectably affect basal PWBC protein-tyrosyl phosphorylation (figure 1C). Subsequently, titration experiments showed that incubation of PWBC for 24 h with 1.5 μM GSAO readily induced hyper-phosphorylation (figure 1D). Similar results were obtained for threonine phosphorylation of proteins upon GSAO treatment of PWBC (additional file 2). Nine different commercial anti-pSer antibodies were also tested, but all were found not to be suitable for our purposes (listed in additional file 9; data not shown).

In a subsequent set of experiments, the effects of GSAO vs. GSCA on specific signalling proteins in PWBC were investigated. The results are described and shown in additional text file and additional files 1 to 11.

From this large data set we conclude that GSAO has rather pleiotropic effects on the cell signalling protein network of PWBC cultured in low serum conditions. Since it is very unlikely that a drug which affects numerous key signalling proteins would show the low toxicity profile seen *in vivo *[10], we modified the PWBC culture system.

### GSAO affects only a small subset of signalling proteins when PWBC are cultured in high serum

**Figure 2:**
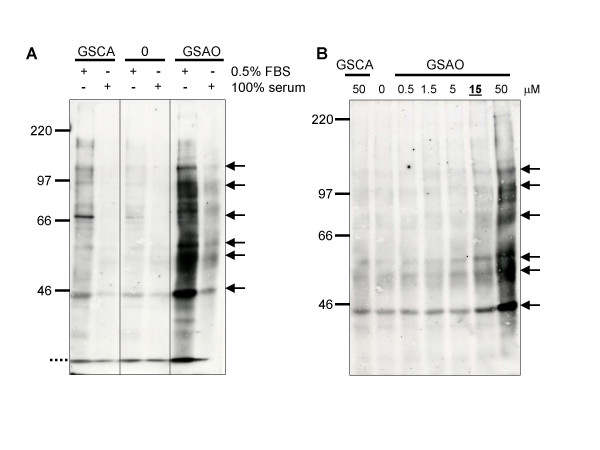
**Effects of GSAO on protein tyrosine phosphorylation are also visible under high serum culture conditions**. **A **PWBC were cultured in low serum (0.5% FBS) or 100% donor serum and treated with 50 μM GSAO or GSCA for 24 h where indicated. Overall, levels of protein tyrosine phosphorylation are lower in donor serum-cultured PWBC and the hyperphosphorylation of tyrosyl residues observed upon GSAO-treatment is less pronounced but still detectable. **B **GSAO dose-response of PWBC cultured in 100% donor serum. PWBC were treated for 24 h. GSAO-induced hyperphosphorylation on tyrosyl residues is clearly visible with 15 μM GSAO.

Figure 2 shows a comparison of GSAO-induced effects on protein tyrosine phosphorylation of PWBC cultured in low serum (0.5% FBS) versus 100% of donor serum. Culture of PWBC in 100% donor serum reduces the overall level of tyrosine phosphorylation in non-treated and GSCA-treated cells. It also reduces the intensity of changes seen upon GSAO-treatment. Titration of GSAO showed that, in the presence of 100% donor serum, increased PWBC tyrosine phosphorylation is clearly observable with 15 μM GSAO treatment of PWBC for 24 h (figure 2B).

**Figure 3:**
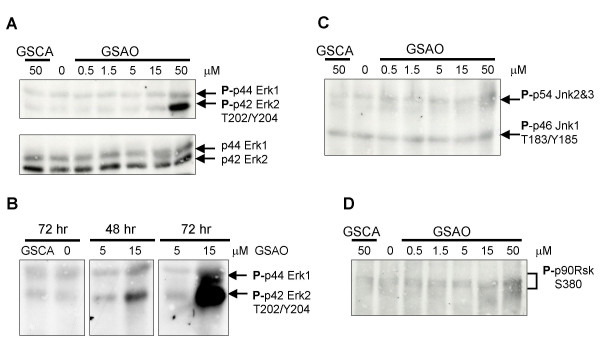
**GSAO effects Erk, but not p90Rsk or Jnk phosphorylation in PWBC high serum culture**. PWBC were cultured in 100% donor serum and treated with GSAO or GSCA for 24 h as indicated.**A **Phosphorylation of Erk2 on the Thr202/Tyr204 epitope is visible at 50 μM GSAO (upper panel). Total Erk protein levels remain unchanged after GSAO treatment for 24 h (lower panel). **B **Phosphorylation of Ser380 on p90Rsk is marginally increased at best in high serum cultured PWBC. **C **Phosphorylation of the Thr183/Tyr185 epitope on Jnks is marginally increased at best in high serum cultured PWBC.

Subsequent analyses focussed on GSAO effects on high serum-cultured PWBC with those phospho-epitope-specific antibodies, which had shown phosphorylation changes under low serum culture conditions. From these experiments, it is obvious that GSAO is affecting only a much smaller number of proteins in high serum culture. For example, p90Rsk phosphorylation on Ser380 and phosphorylation of JNKs is at best barely observable in high serum culture (figure 3C and 3D; compare to additional files 3 and 5). However, phosphorylation of Erk2 on Thr202/Tyr204 is obvious after exposure of cells to 50 μM GSAO for 24 h (figure 3A). Because the trial will involve infusions of GSAO, more chronic exposure was studied. Extension of the GSAO exposure time on PWBC in high serum culture showed that 15 μM GSAO induces Erk2 activation after 48 h and after 72 h a massive activation of Erk2 is seen (figure 3B).

**Figure 4:**
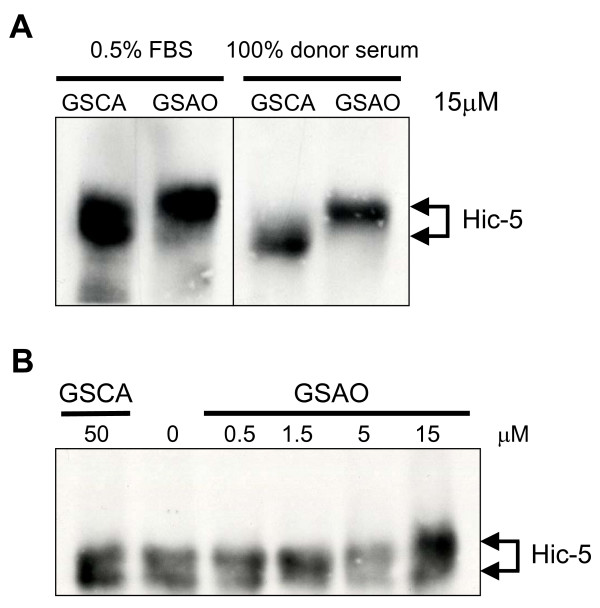
**Prominent mobility change of Hic-5 in GSAO-treated PWBC in low and high serum culture**. Cells were treated with GSCA or GSAO as indicated for 24 h and analysed for Hic-5 expression and mobility. **A **Comparisonof effects induced by 15 μM GSAO under low and high serumculture conditions. In both cases a prominent band shift of Hic-5 isobserved. **B **GSAO dose – response of Hic-5 in PWBC culturedin 100% donor serum. A partial mobility shift is observable at 5 μM GSAO, but the band shift is more pronounced at 15 μM GSAO.

The paxillin homologue Hic-5 (hydrogen oxide inducible clone-5; [14] was one of the few other proteins which was clearly affected by GSAO under high serum culture conditions (figure 4). GSAO treatment induces a massive mobility shift of the Hic-5 band at a concentration of 15 μM compound. This upshift is probably a consequence of an altered phosphorylation state of Hic-5, however, other modifications of Hic-5 upon GSAO treatment of PWBC cannot be excluded. Phosphorylation of Hic-5 on Tyr38 and 60 has been previously reported [15,16], but phospho-specific antibodies for these epitopes are unavailable. A slight shift in Hic-5 mobility was occasionally seen with as little as 5 μM GSAO (figure 4B).

**Figure 5:**
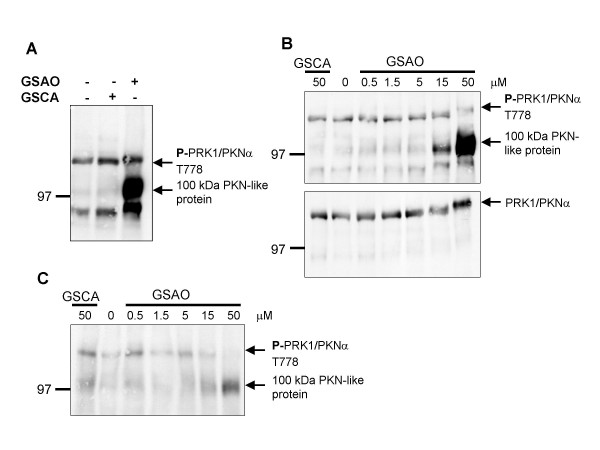
**GSAO induces phosphorylation of a 100 kDa protein which is recognised by an antibody directed against PRK1/PKNα pThr778**. PWBC were treated with GSCA or GSAO where indicated for 24 h and proteins analysed with anti-PRK1/PKNα pThr778. **A **Effect of 50 μM GSAOvs. GSCA on a 100 kDa protein recognised by anti-PRK1/PKNα pThr 778. A prominent hyperphosphorylation of the 100 kDa protein is visible, while a signal of ca. 120 kDa, the size expected for PRK1/PKNα, remains unchanged. The 100 kDa band may correspond to the PRK1/PKNα relative PKNβ, but this can only be verified after the generation of PKNβ-specific antibodies. **B **GSAO dose – response in low serum cultured PWBC. Hyperphosphorylation of the 100 kDa band is visible from 15 μM of GSAO with the pThr778-specific PRK1/PKNα antibody (upper panel) while total protein levels of PRK1/PKNα remain unchanged (lower panel). A mobility shift of PRK1/PKNα is also visible with this antibody at higher GSAO concentrations, possibly indicating a PRK1/PKNα phosphorylation at a site different from pThr778. **C **Effect of GSAO on the 100 kDa band in high serum cultured PWBC. A moderate increase of phosphorylation at the pThr778-related epitope is visible at 50 μM GSAO.

A third protein clearly affected by GSAO in high serum PWBC culture was a PRK1/PKNα antibody-immunoreactive band of 100 kDa (figure 5). Protein kinase C-related kinases (PRKs, also designated PKNs) are known effectors of Rho GTPases and activated by fatty acids and lipids in vitro [17]. Phosphorylation of PRK1/PKNα Thr778 (corresponding to PRK2/PKNγ Thr816) in the activation loop of the kinase leads to an increased catalytic activity of PRKs/PKNs. While PRK1/PKNα and PRK2/PKNγ are ca. 120 and 140 kDa in size, respectively, PKNβ has an apparent molecular weight of ca. 100 kDa. We were so far unable to determine whether the observed band is indeed PKNβ, due to a lack of suitable specific reagents.

In summary, the screen with a large number of epitope-specific antibodies has resulted in the detection of three candidate surrogate markers for GSAO activity in PWBC under high serum conditions. To search for further potential markers, antibodies targeted against phosphorylated substrate epitopes or docking motifs of a specific kinase or kinase family were used on GSAO-, GSCA- or mock-treated PWBC lysates.

### Detection of further proteins affected by GSAO in PWBC high serum culture using kinase substrate-specific antibodies

**Figure 6:**
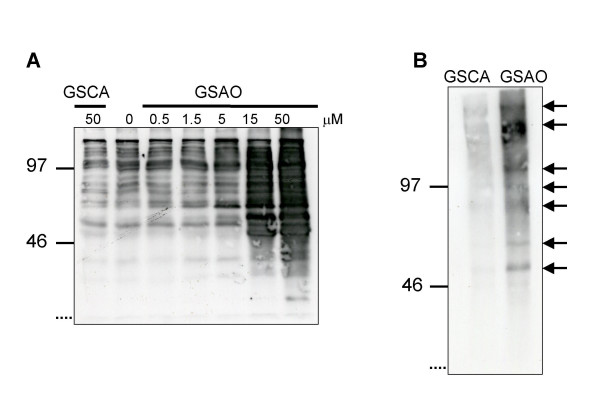
**Detection of proteins hyperphosphorylated after treatment of PWBC with GSAO using an Akt substrate-directed antibody**. Cells were incubated for 24 hwith GSAO or GSCA as indicated and analysed with an antibody made to recognise multiple Akt substrate proteins in their Akt-phosphorylated forms (R/K-x-R/K-x-x-pS/pT motif, Cell Signalling Technology 9611). **A **Massive effect of GSAO on phosphoproteins detected with the Akt substrate phospho-antibody using protein extracts from low serum cultured PWBC. The control compound GSCA does not detectably change the basal phospho-protein pattern. **B **Effects of 15 μM GSAO on high serum culturedPWBC. Arrows indicate bands that are hyperphosphorylated inGSAO-treated cells.

Phosphorylated substrate epitope-directed antibodies for the protein targets of the kinase families Akt, ATM/ATR, Cdk, MAPK, PKA, PKC and PKD, as well as a PDK-1 phospho-docking motif antibody (for details see additional file 9) were used to detect changes in kinase substrate proteins of PWBC cultured in high or low serum in the presence or absence of GSAO and GSCA. Selected results are shown in figures 6 and additional files 6 and 7. GSAO-induced protein phosphorylation changes were again, as expected, much more pronounced under low serum culture conditions and served as a useful control for the general functionality of the antibodies. GSAO-effects in high serum-cultured PWBC were observed with the Akt (figure 6), PKA (additional file 6) and PKD (additional file 7) substrate-directed antibodies. These results show that a range of further proteins affected by GSAO-exposure of PWBC cultured in high serum remain to be identified.

### GSAO affects signalling proteins in HUVEC endothelial cells

**Figure 7:**
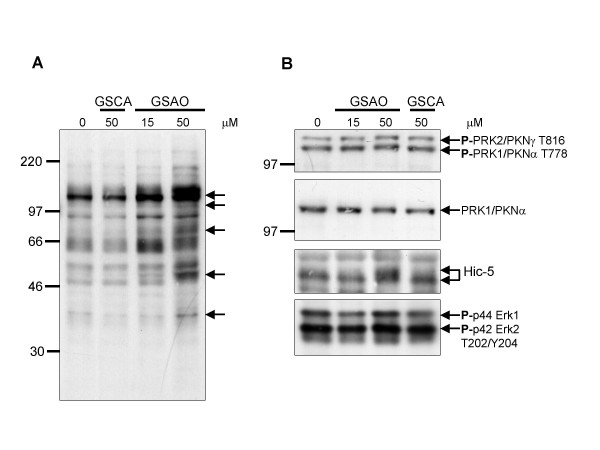
**Effects of GSAO treatment on HUVEC**. HUVEC wereincubated for 24 h with GSAO or GSCA as indicated with 15% FBS. **A **Increased tyrosine phosphorylation is readily detectable at 15 μM GSAO treatment. Some GSAO affected proteins are indicated witharrows. **B **Hyperphosphorylation of the 100 kDa protein is undetectable in HUVEC, while a band of ca. 120 kDa, the size expected for PRK1/PKNα, remains unchanged. A band shift of Hic-5 is detectable at 50 μM GSAO treatment. Phosphorylation changes in Erk2 at Thr202/Tyr204 are not detectable.

GSAO targets proliferating endothelial cells *in vivo *[10], therefore experiments were carried out with primary endothelial cells. Human umbilical vein endothelial cells (HUVEC) were treated with 15 or 50 μM GSAO. Increased tyrosine phosphorylation was observed in the HUVEC upon 15 μM treatment with GSAO (figure 7A). The PRK1/PKNα Thr778 antibody-immunoreactive band of 100 kDa was not detectable in HUVEC upon treatment with GSAO and no changes in phosphorylation or protein expression of PRK1/PKNα were detectable (figure 7B). No induction of phosphorylation of Erk1/2 Thr202/Tyr204 was observed (figure 7B). However, GSAO treatment of HUVEC with 50 μM GSAO clearly induces a mobility shift of the Hic-5 band (see figure 7B) similar to that seen in PWBC cultured in high serum.

## Discussion

STM drugs are playing an increasingly important role in cancer therapy. Many of these drugs appear to have low toxicity profiles, so biological responses are expected below drug concentrations, which lead to massive side effects. Based on the results from previous animal studies [10,18], one can speculate that this may also be the case for novel anti-cancer compound GSAO. Surrogate markers that can be monitored to detect the biological activity of a STM drug in patients shortly after drug application may therefore be of great value for its optimal clinical development.

GSAO is thought to primarily interfere with proliferating endothelial cells [10], hence disrupting the further growth of tumour vasculature. GSAO activity is therefore dependent on systemic distribution through the blood vessels and capillaries but probably does not need to penetrate deeply into tissues to exert key effects. As endothelial cells and PWBC are both key components of the blood vessel system and will be exposed to circulating GSAO to a similar degree, PWBC should be well suited for the analysis of surrogate markers for GSAO activity *in vivo*.

Our experiments clearly show that classical low serum culture, often used to reduce background signals prior to addition of a drug or factor, is not ideal to find potential markers for GSAO bioactivity *in vivo*. Furthermore, this study was only possible through the multitude of commercial signalling protein antibodies that have become available in the last few years. These antibodies, directed against specific phosphorylated or nonphosphorylated protein epitopes in signalling proteins, allow the rapid analysis of a significant part of the signalling phosphoproteome. In order to obtain reliable data with these antibodies, their specificity must be carefully evaluated. In our western blot experiments, we frequently found multiple bands resulting from a single antibody analysed. Signals appeared often at unexpected sizes, especially for polyclonal anti sera. Until now, high quality monoclonal antibodies are only available for a small number of phosphoepitopes. We feel that this would make, at present, results from high throughput (HTP) array-format assays or FACS-based HTP analyses like multiplex-bead assays difficult to properly evaluate.

Monitoring of drug effects on total protein expression levels, is simpler, but appears to be overall less informative as many phospho-changes are not mirrored by changes in overall protein expression. Nevertheless, it can give some initial leads and many good quality monoclonal antibodies are readily available. For optimal retrieval of information from western blot type assays it is necessary to perform non-automated visual re-inspections to detect all changes. The GSAO-induced Hic-5 mobility shift was found through this approach and would have been missed using a spot array or another technique not separating proteins by apparent molecular weight.

Hic-5 is an interesting candidate for further analyses of GSAO actions in cells. It is a member of the paxillin family [19,20] and has been implicated in a variety of biological processes including those that regulate cell motility, invasion, survival and proliferation (summarised in [19]). Moreover, recent publications have highlighted additional roles for Hic-5, for example, the suppression of Lef/Tcf-driven transcription during vertebrate development [21]. Hic-5 was also reported to undergo redox-sensitive nuclear-cytoplasmic shuttling [22,23] and to interact with Traf4, which is implicated in the oxidative regulation of endothelial cell migration [24]. GSAO uptake and action has also been shown to depend on redox parameters like cellular glutathione levels [11], but it remains to be studied if GSAO directly influences theredox regulation of cells or cellular subcompartments, for example by interfering with redox-regulated phosphatases like PTP-PEST [24] or other signalling proteins.

The consistent activation of the MAP kinase Erk2 by GSAO under high and low serum conditions is also intriguing, since no activation of its regulator kinases MEK1/2 could be observed (data not shown). Apart from a direct effect on Erk-inactivating phosphatases, like MKPs, other more indirect changes in PWBC signalling may lead to Erk2 activation. Since the intracellular GSAO uptake is very slow and effects are usually seen after many hours, a plethora of possible indirect mechanisms, including changes in the transcription of certain genes may underlie the observed Erk2 activity change. Erk2 activation is thought to trigger its dimerisation and nuclear translocation [25], possibly leading to the phosphorylation of nuclear substrate proteins. Analyses of selected Erk substrates like p90Rsk, and Elk-1 with phospho-epitope specific antibodies (results summarised in additional file 9) did not pinpoint an affected Erk2 substrate so far and an initial screen for substrates of proline-directed kinases (like Erks and Cdks) with an antibody which recognises an xxx-pThr-Pro-xxx motif did also not detect candidate substrates in GSAO-treated PWBC culture in high serum (not shown). Since more than 70 Erk substrates have been reported so far, a large number of potential substrates remain to be analysed. Beyond this, experiments with substrate epitope-directed phospho-antibodies specific for targets of other kinases clearly indicate that additional GSAO-affected proteins, which could be useful bioactivity markers, remain to be discovered.

Changes in one of the three candidate surrogate markers for GSAO activity found in PWBC is detectable in endothelial cells, but additional changes in tyrosine phosphorylation were observed in a multitude of proteins at 15 μM GSAO treatment in HUVEC. This suggests that overlapping, but distinct groups of signalling pathways are affected by GSAO in the different cell types. Further studies into the biological mechanisms of GSAO interference with endothelial cell proliferation are needed to elucidate the *in vivo *activity of GSAO in this cell type.

## Conclusion

This study has shown that GSAO effects on the PWBC phosphoprotein signalling network are quite selective, provided the appropriate experimental conditions are used. The results also give suggestions for the development of new reagents, which may be useful to support future clinical studies and to possibly gain further insight into the cellular targets of GSAO. Analyses with substrate epitope-specific antibodies show that further GSAO-affected proteins remain to be identified. Variation in phosphorylation patterns between PWBC and HUVEC suggest distinct pathway patterns become activated by GSAO in different cell types. As PWBC are easily obtainable they are more probably suitable for measuring surrogate markers in future clinical studies, but endothelial cells are clearly also an important cell type that needs to be analysed further with molecular and functional assays.

## Competing interests

All authors declare not to have any competing interests. Cancer Research UK, which funds the work of VAC, SMF and ALH, is a sponsor and organiser of the planned GSAO clinical trial.

## Authors' contributions

VAC performed most of the experiments and participated in the drafting of the manuscript. PJH provided the GSCA and GSAO and critically commented on the drafted manuscript. ALH participated in the design of some experiments and critically commented on the drafted manuscript. SMF designed the study, drafted the manuscript and performed some initial experiments.

## Pre-publication history

The pre-publication history for this paper can be accessed here:



## Supplementary Material

Additional File 1 **GSAO and phenylarsine oxide (PAO) induce similar patterns of tyrosine phosphorylation in peripheral white blood cells (PWBC)**. Freshly isolated PWBC were equilibrated in medium with 0.5% FBS for 2 h and then treated with the indicated concentrations of PAO or GSAO. PWBC were subsequently lysed and proteins analysed for protein tyrosine phosphorylation by western blot. Uptake of the hydrophilic GSAO into cells is, as expected, very slow. Treatment of PWBC with 50 μM GSAO for 8 h results in protein tyrosyl-hyperphosphorylation similar to that seen upon incubation with 1 μM PAO for 1 h. Hyperphosphorylated protein bands are indicated by arrows. Click here for file

Additional File 2 **Dose-dependent increase of PWBC protein threonine phosphorylation induced by GSAO**. PWBC were treated with GSAO or GSCA where indicated for 24 h and threonine phosphorylation detected by western blot. **A **50 μM GSCA does not alter the protein pThr levels of PBWC detectably. **B **Changes in protein pThr patterns are apparent with as little as 1.5 μM GSAO. GSAO-affected proteins are indicated with arrows. Click here for file

Additional File 3 **GSAO induces phosphorylation of c-Jun N-terminal kinases (JNKs) at the kinase activity-regulating epitope (Thr 183/Tyr 185) in PWBC**. PWBC were treated with GSAO or GSCA, then lysed and analysed by western blot for JNK expression or phosphorylation. The phospho-specific antibody detects JNK protein only effectively when doubly phosphorylated on Thr183 and Tyr185. **A **Cells were treated with 50 μM GSAO or GSCA for 24 h where indicated and analysed for phospho-JNK (upper panel) or total JNK (lower panel) **B **Cells were treated with different concentrations of GSAO for 24 h as indicated and analysed for phospho-JNK. Click here for file

Additional File 4 **Effects of GSAO on the MAP kinase cascade**. Cells were treated with GSAO or GSCA as indicated for 24 h and lysates analysed for the phosphorylation of Erk1/Erk2 (pThr202/pTyr204) or c-Raf-1 (pTyr340/pTyr341; pSer259). Phosphorylation of Erk1/Erk2 at the epitope Thr202/Tyr204 correlates closely with Erk kinase activation. The phospho-epitopes analysed for c-Raf-1 also contribute to the regulation of c-Raf-1 kinase activity, but numerous other regulatory sites have been described in c-Raf-1. **A **Effect of GSAO treatment on the phosphorylation of Erk1/Erk2 (pThr202/pTyr204) detected by a polyclonal antibody (9101, Cell Signalling Technology). Apart from the major Erk1/2 phospho-bands, several additional phospho-bands of different sizes, some possibly representing other Erk family members, are visible. **B **Effect of GSAO treatment on the phosphorylation of Erk1/Erk2 (pThr 202/pTyr204) detected by a monoclonal antibody (M8159, Sigma). **C **Effect of GSAO treatment on the phosphorylation of c-Raf-1 at Tyr340/Tyr341. Phosphorylation of this epitope can contribute to the activation of Raf kinase. **D **Effect of GSAO treatment on the phosphorylation of c-Raf-1 at Ser259. Dephosphorylation of this epitope is thought to be important for activation of the kinase. The c-Raf-1 band shift observed with 15 and 50 μM GSAO, probably resulting from phosphorylation events at other c-Raf-1 epitopes, makes it difficult to determine if a partial dephosphorylation of pSer259 occurs at these GSAO concentrations. Click here for file

Additional File 5 **Effects of GSAO on the phosphorylation of p90Rsk at Ser380 in PWBC obtained from different volunteers**. PWBC were treated with GSAO or GSCA as indicated for 24 h and lysates analysed for the phosphorylation of Ser380 in p90Rsk. Cells from three different donors were used. The male donor was analysed on two different occasions. Increased Ser380 phosphorylation is clearly visible at 5 μM GSAO in all cases. With 50 μM GSAO a major mobility shift of p90Rsk (pSer380) is visible. This could indicate the occurrence of additional phosphorylation events. Click here for file

Additional File 6 **Detection of proteins hyperphosphorylated after treatment of PWBC with GSAO using a PKA substrate-directed antibody**. Cells were incubated for 24 h with GSAO or GSCA as indicated and analysed with an antibody made to recognise multiple PKA substrate proteins in their PKA-phosphorylated forms (R-R-x-pS or R-x-x-pT motif, Cell Signalling Technology 9621). **A **Strong effect of GSAO on phosphoproteins detected with the PKC substrate phospho-antibody using protein extracts from low serum cultured PWBC. The control compound GSCA does not detectably change the basal phospho-protein pattern. **B **Effects of 15 μM GSAO on high serum cultured PWBC. Arrows indicate bands that are slightly hyperphosphorylated in GSAO-treated cells. Click here for file

Additional File 7 **Detection of proteins hyper-phosphorylated after treatment of PWBC with GSAO using a PKD substrate-directed antibody**. Cells were incubated for 24 h with GSAO or GSCA as indicated and analysed with an antibody made to recognise multiple PKD substrate proteins in their PKD-phosphorylated forms (L-x-R-x-x-pT/pS motif, Cell Signalling Technology 4381) **A **Effect of GSAO on phosphoproteins detected with the PKC substrate phospho-antibody using protein extracts from low serum cultured PWBC. The control compound GSCA does not detectably change the basal phospho-protein pattern. **B **Effects of 15 μM GSAO on high serum cultured PWBC. Arrows indicate three bands that are clearly hyperphosphorylated in GSAO-treated cells. Click here for file

Additional File 8 **Compiled results of the Powerblot™ comparison of PWBC cultured in low serum and treated with 50 μM GSAO or GSCA.** Click here for file

Additional File 9 **Antibodies used for analysis of GSAO effects on PWBC.** Click here for file

Additional File 10 **Summary of GSAO-induced protein expression changes in PWBC cultured with low serum medium**. Of 240 individually selected signalling proteins, which were analysed by the commercially available Powerblot™, the 24 proteins listed here showed an apparent change in protein expression considered to be highly significant according to the company. Proteins in blue color are upregulated, proteins in black downregulated with GSAO vs. GSCA. From this list it is obvious, that kinases and PTPases and their targets make up the majority of proteins affected in their expression level by GSAO. At least two of the signals (LAT, VASP) are, however, clearly questionable, as the detected band size in the sample varies greatly from the expected protein size. In other cases, for example Hic-5, an expression change is detected by the automated system. Upon visual inspection this supposed expression change results from a mobility shift of Hic 5. A full list of all proteins analysed and changes detected are compiled in additional file 8. Click here for file

Additional File 11 **Pleiotropic effects on PWBC in low serum conditions.** Click here for file

## References

[B1] Alonso A, Sasin J, Bottini N, Friedberg I, Osterman A, Godzik A, Hunter T, Dixon J, Mustelin T (2004). Protein tyrosine phosphatases in the human genome. Cell.

[B2] Andersen JN, Jansen PG, Echwald SM, Mortensen OH, Fukada T, Del Vecchio R, Tonks NK, Moller NP (2004). A genomic perspective on protein tyrosine phosphatases: gene structure, pseudogenes, and genetic disease linkage. Faseb J.

[B3] Wang Z, Shen D, Parsons DW, Bardelli A, Sager J, Szabo S, Ptak J, Silliman N, Peters BA, van der Heijden MS, Parmigiani G, Yan H, Wang TL, Riggins G, Powell SM, Willson JK, Markowitz S, Kinzler KW, Vogelstein B, Velculescu VE (2004). Mutational analysis of the tyrosine phosphatome in colorectal cancers. Science.

[B4] Ducruet AP, Vogt A, Wipf P, Lazo JS (2005). Dual specificity protein phosphatases: therapeutic targets for cancer and Alzheimer's disease. Annu Rev Pharmacol Toxicol.

[B5] Gasparotto D, Maestro R, Piccinin S, Vukosavljevic T, Barzan L, Sulfaro S, Boiocchi M (1997). Overexpression of CDC25A and CDC25B in head and neck cancers. Cancer Res.

[B6] Guo J, Kleeff J, Li J, Ding J, Hammer J, Zhao Y, Giese T, Korc M, Buchler MW, Friess H (2004). Expression and functional significance of CDC25B in human pancreatic ductal adenocarcinoma. Oncogene.

[B7] Pestell KE, Ducruet AP, Wipf P, Lazo JS (2000). Small molecule inhibitors of dual specificity protein phosphatases. Oncogene.

[B8] Cristofanilli M, Charnsangavej C, Hortobagyi GN (2002). Angiogenesis modulation in cancer research: novel clinical approaches. Nat Rev Drug Discov.

[B9] Donoghue N, Yam PT, Jiang XM, Hogg PJ (2000). Presence of closely spaced protein thiols on the surface of mammalian cells. Protein Sci.

[B10] Don AS, Kisker O, Dilda P, Donoghue N, Zhao X, Decollogne S, Creighton B, Flynn E, Folkman J, Hogg PJ (2003). A peptide trivalent arsenical inhibits tumor angiogenesis by perturbing mitochondrial function in angiogenic endothelial cells. Cancer Cell.

[B11] Dilda PJ, Don AS, Tanabe KM, Higgins VJ, Allen JD, Dawes IW, Hogg PJ (2005). Mechanism of selectivity of an angiogenesis inhibitor from screening a genome-wide set of Saccharomyces cerevisiae deletion strains. J Natl Cancer Inst.

[B12] Defilippi P, Retta SF, Olivo C, Palmieri M, Venturino M, Silengo L, Tarone G (1995). p125FAK tyrosine phosphorylation and focal adhesion assembly: studies with phosphotyrosine phosphatase inhibitors. Exp Cell Res.

[B13] Oetken C, von Willebrand M, Autero M, Ruutu T, Andersson LC, Mustelin T (1992). Phenylarsine oxide augments tyrosine phosphorylation in hematopoietic cells. Eur J Haematol.

[B14] Shibanuma M, Mashimo J, Kuroki T, Nose K (1994). Characterization of the TGF beta 1-inducible hic-5 gene that encodes a putative novel zinc finger protein and its possible involvement in cellular senescence. J Biol Chem.

[B15] Hetey SE, Lalonde DP, Turner CE (2005). Tyrosine-phosphorylated Hic-5 inhibits epidermal growth factor-induced lamellipodia formation. Exp Cell Res.

[B16] Ishino M, Aoto H, Sasaski H, Suzuki R, Sasaki T (2000). Phosphorylation of Hic-5 at tyrosine 60 by CAKbeta and Fyn. FEBS Lett.

[B17] Mukai H (2003). The structure and function of PKN, a protein kinase having a catalytic domain homologous to that of PKC. J Biochem (Tokyo).

[B18] Dilda PJ, Decollogne S, Rossiter-Thornton M, Hogg PJ (2005). Para to ortho repositioning of the arsenical moiety of the angiogenesis inhibitor 4-(N-(S-glutathionylacetyl)amino)phenylarsenoxide results in a markedly increased cellular accumulation and antiproliferative activity. Cancer Res.

[B19] Brown MC, Turner CE (2004). Paxillin: adapting to change. Physiol Rev.

[B20] Turner CE (2000). Paxillin and focal adhesion signalling. Nat Cell Biol.

[B21] Ghogomu SM, Vanvenrooy S, Ritthaler M, Wedlich D, Gradl D (2005). Hic-5, a novel repressor of Lef/Tcf driven transcription. J Biol Chem.

[B22] Shibanuma M, Kim-Kaneyama JR, Ishino K, Sakamoto N, Hishiki T, Yamaguchi K, Mori K, Mashimo J, Nose K (2003). Hic-5 communicates between focal adhesions and the nucleus through oxidant-sensitive nuclear export signal. Mol Biol Cell.

[B23] Shibanuma M, Mori K, Kim-Kaneyama JR, Nose K (2005). Involvement of FAK and PTP-PEST in the regulation of redox-sensitive nuclear-cytoplasmic shuttling of a LIM protein, Hic-5. Antioxid Redox Signal.

[B24] Wu RF, Xu YC, Ma Z, Nwariaku FE, Sarosi GAJ, Terada LS (2005). Subcellular targeting of oxidants during endothelial cell migration. J Cell Biol.

[B25] Cobb MH, Goldsmith EJ (2000). Dimerization in MAP-kinase signaling. Trends Biochem Sci.

