# Supplementary figures and images for: Molecular profiling of signalling proteins for effects induced by the anti-cancer compound GSAO with 400 antibodies
Source: BMC Cancer. 2006 Jun 9;6:155. doi: 10.1186/1471-2407-6-155 (PMC1550423; doi:10.1186/1471-2407-6-155)

## Slide 1
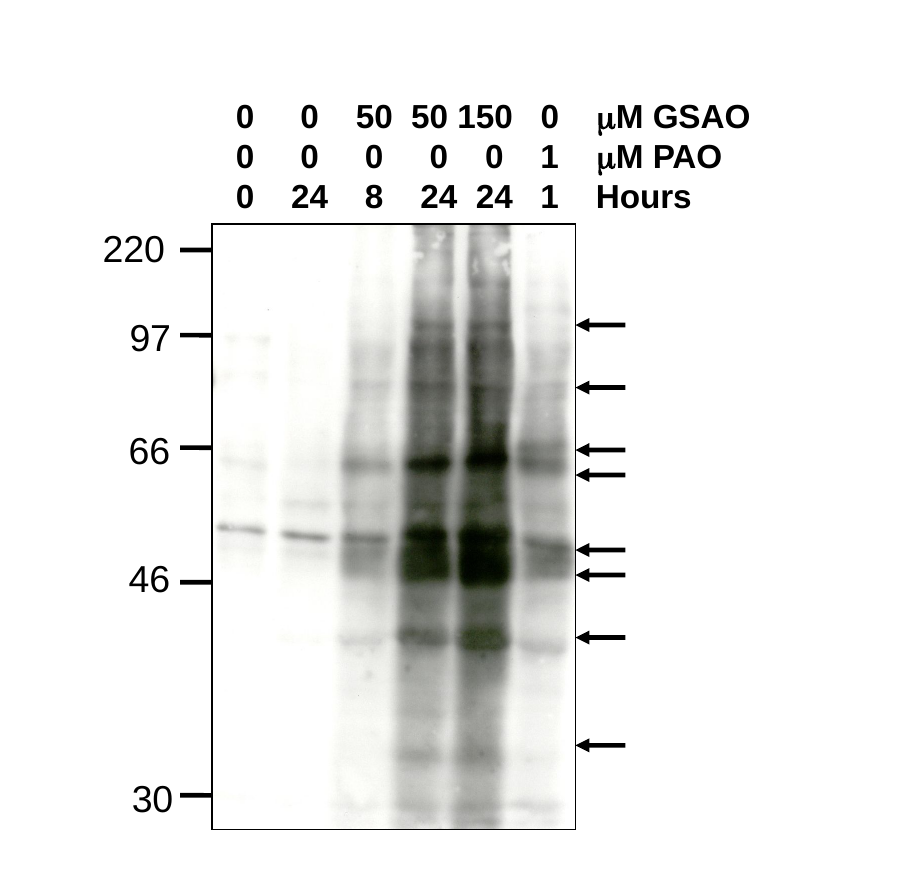

0 0 50 50 150 0 μM GSAO
 0 0 0 0 0 1 μM PAO
 0 24 8 24 24 1 Hours
220
97
66
46
30

Supplement: Additional File 1 — GSAO and phenylarsine oxide (PAO) induce similar patterns of tyrosine phosphorylation in peripheral white blood cells (PWBC). Freshly isolated PWBC were equilibrated in medium with 0.5% FBS for 2 h and then treated with the indicated concentrations of PAO or GSAO. PWBC were subsequently lysed and proteins analysed for protein tyrosine phosphorylation by western blot. Uptake of the hydrophilic GSAO into cells is, as expected, very slow. Treatment of PWBC with 50 μM GSAO for 8 h results in protein tyrosyl-hyperphosphorylation similar to that seen upon incubation with 1 μM PAO for 1 h. Hyperphosphorylated protein bands are indicated by arrows. [file 1471-2407-6-155-S1.PPT]

## Slide 1
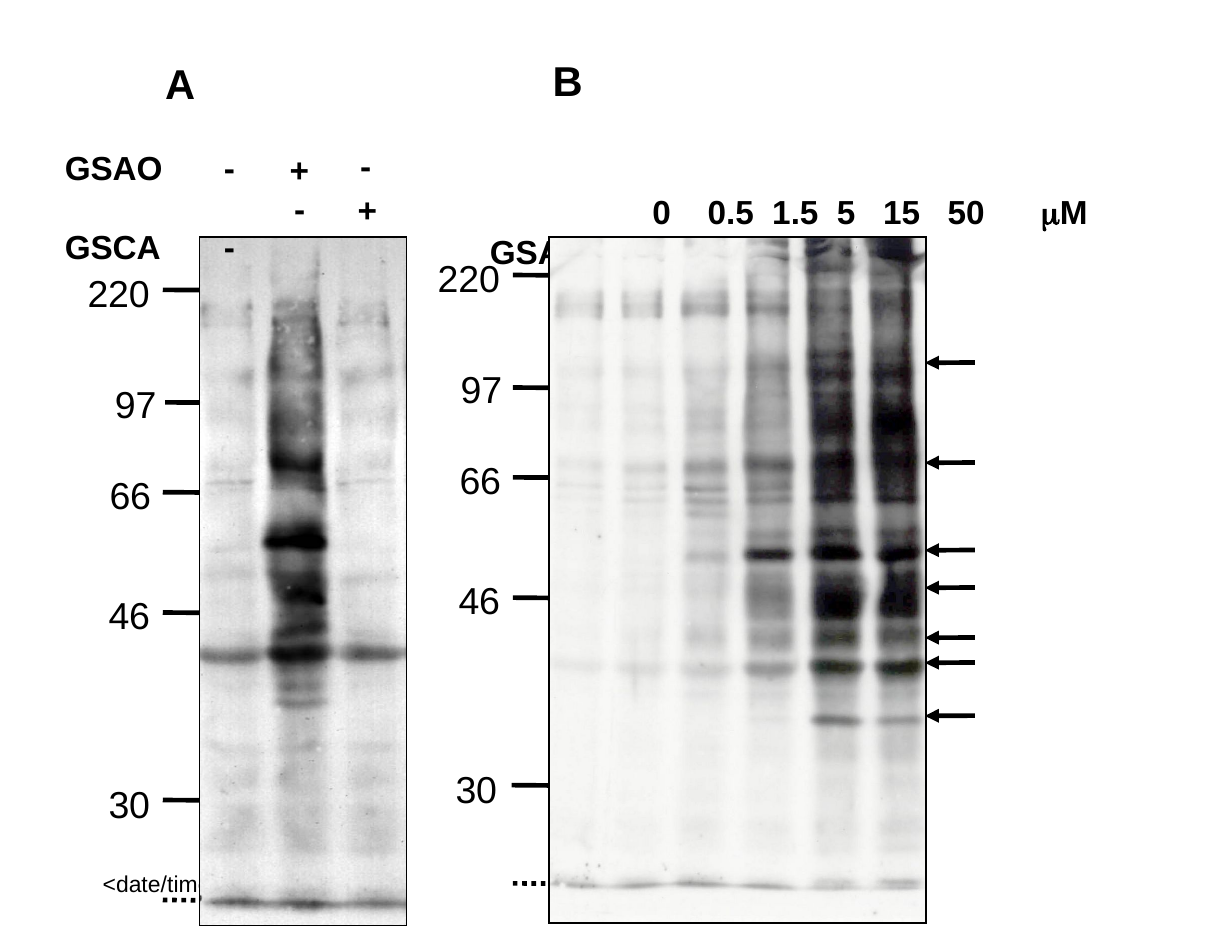

B
A
-
GSAO	 -
GSCA	 -
+
-
+
 0 0.5 1.5 5 15 50 μM GSAO
220
220
97
97
66
66
46
46
30
30
<date/time>
<footer>

Supplement: Additional File 2 — Dose-dependent increase of PWBC protein threonine phosphorylation induced by GSAO. PWBC were treated with GSAO or GSCA where indicated for 24 h and threonine phosphorylation detected by western blot. A 50 μM GSCA does not alter the protein pThr levels of PBWC detectably. B Changes in protein pThr patterns are apparent with as little as 1.5 μM GSAO. GSAO-affected proteins are indicated with arrows. [file 1471-2407-6-155-S2.PPT]

## Slide 1
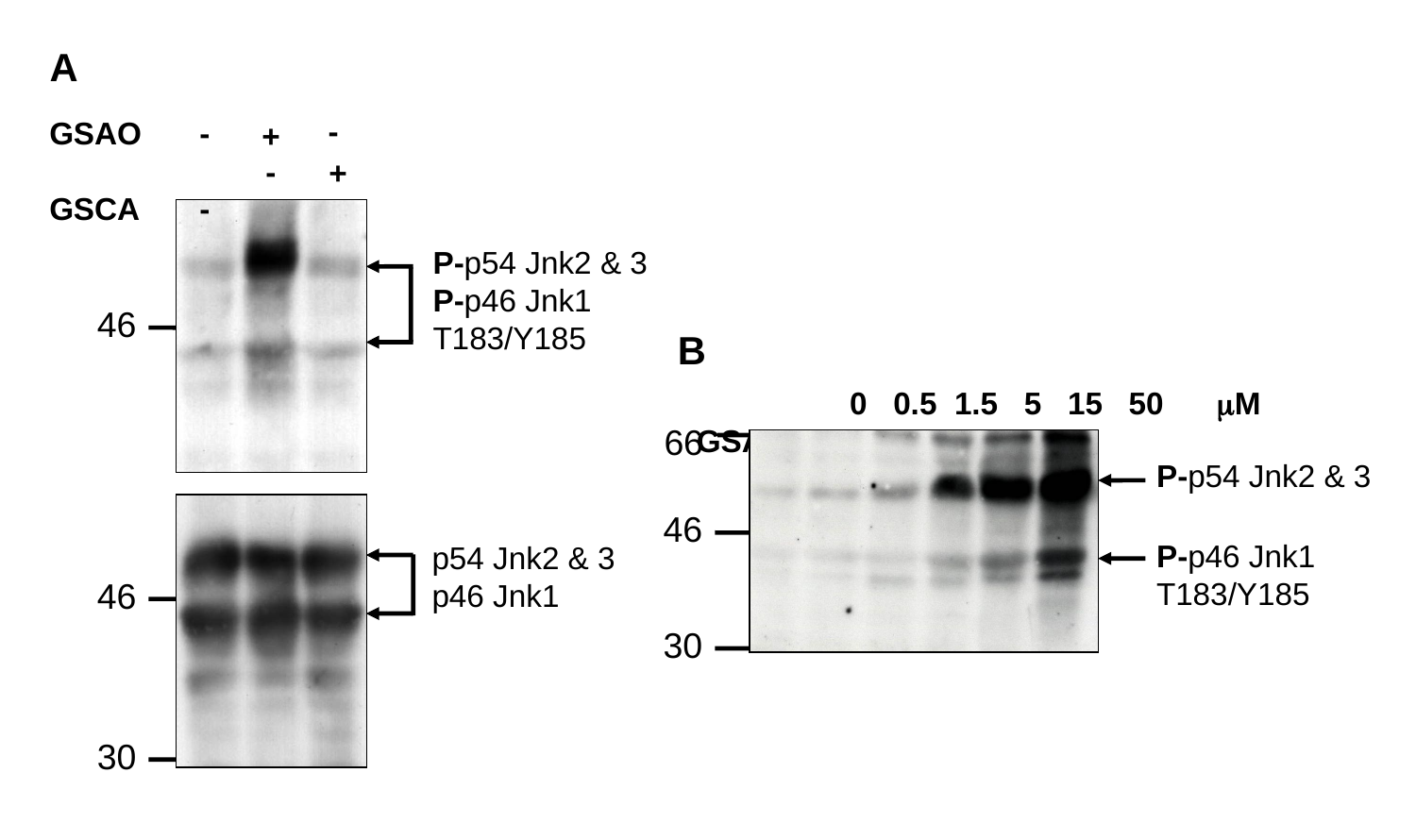

A
-
GSAO	 -
GSCA	 -
+
-
+
P-p54 Jnk2 & 3
P-p46 Jnk1
T183/Y185
46
B
 0 0.5 1.5 5 15 50 μM GSAO
66
P-p54 Jnk2 & 3
46
P-p46 Jnk1
T183/Y185
p54 Jnk2 & 3
p46 Jnk1
46
30
30

Supplement: Additional File 3 — GSAO induces phosphorylation of c-Jun N-terminal kinases (JNKs) at the kinase activity-regulating epitope (Thr 183/Tyr 185) in PWBC. PWBC were treated with GSAO or GSCA, then lysed and analysed by western blot for JNK expression or phosphorylation. The phospho-specific antibody detects JNK protein only effectively when doubly phosphorylated on Thr183 and Tyr185. A Cells were treated with 50 μM GSAO or GSCA for 24 h where indicated and analysed for phospho-JNK (upper panel) or total JNK (lower panel) B Cells were treated with different concentrations of GSAO for 24 h as indicated and analysed for phospho-JNK. [file 1471-2407-6-155-S3.ppt]

## Slide 1
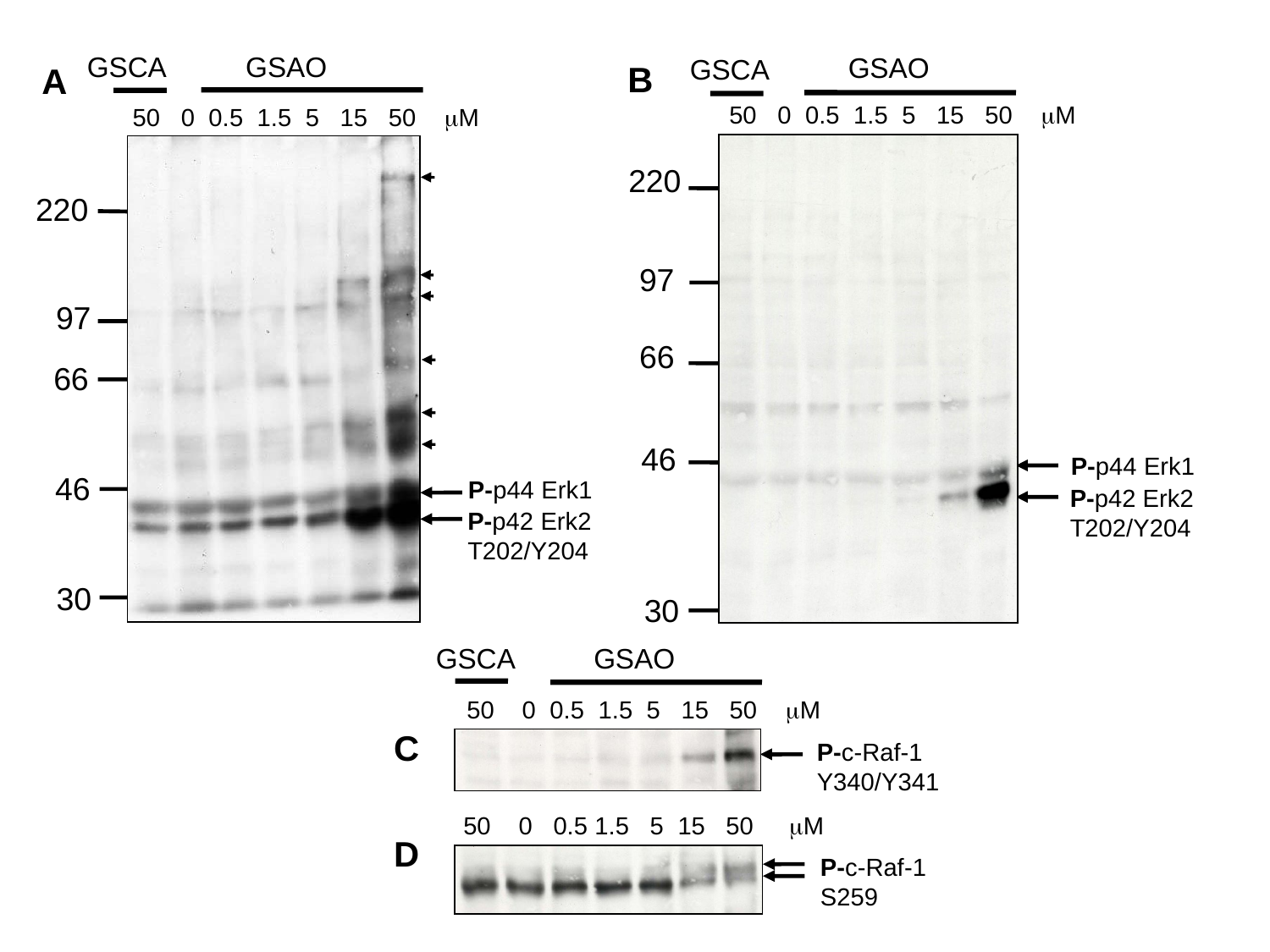

GSCA
GSAO
GSAO
GSCA
B
A
 50 0 0.5 1.5 5 15 50 μM
 50 0 0.5 1.5 5 15 50 μM
220
220
97
97
66
66
46
P-p44 Erk1
46
P-p44 Erk1
P-p42 Erk2
T202/Y204
P-p42 Erk2
T202/Y204
30
30
GSCA
GSAO
 50 0 0.5 1.5 5 15 50 μM
C
P-c-Raf-1
Y340/Y341
 50 0 0.5 1.5 5 15 50 μM
D
P-c-Raf-1
S259

Supplement: Additional File 4 — Effects of GSAO on the MAP kinase cascade. Cells were treated with GSAO or GSCA as indicated for 24 h and lysates analysed for the phosphorylation of Erk1/Erk2 (pThr202/pTyr204) or c-Raf-1 (pTyr340/pTyr341; pSer259). Phosphorylation of Erk1/Erk2 at the epitope Thr202/Tyr204 correlates closely with Erk kinase activation. The phospho-epitopes analysed for c-Raf-1 also contribute to the regulation of c-Raf-1 kinase activity, but numerous other regulatory sites have been described in c-Raf-1. A Effect of GSAO treatment on the phosphorylation of Erk1/Erk2 (pThr202/pTyr204) detected by a polyclonal antibody (9101, Cell Signalling Technology). Apart from the major Erk1/2 phospho-bands, several additional phospho-bands of different sizes, some possibly representing other Erk family members, are visible. B Effect of GSAO treatment on the phosphorylation of Erk1/Erk2 (pThr 202/pTyr204) detected by a monoclonal antibody (M8159, Sigma). C Effect of GSAO treatment on the phosphorylation of c-Raf-1 at Tyr340/Tyr341. Phosphorylation of this epitope can contribute to the activation of Raf kinase. D Effect of GSAO treatment on the phosphorylation of c-Raf-1 at Ser259. Dephosphorylation of this epitope is thought to be important for activation of the kinase. The c-Raf-1 band shift observed with 15 and 50 μM GSAO, probably resulting from phosphorylation events at other c-Raf-1 epitopes, makes it difficult to determine if a partial dephosphorylation of pSer259 occurs at these GSAO concentrations. [file 1471-2407-6-155-S4.ppt]

## Slide 1
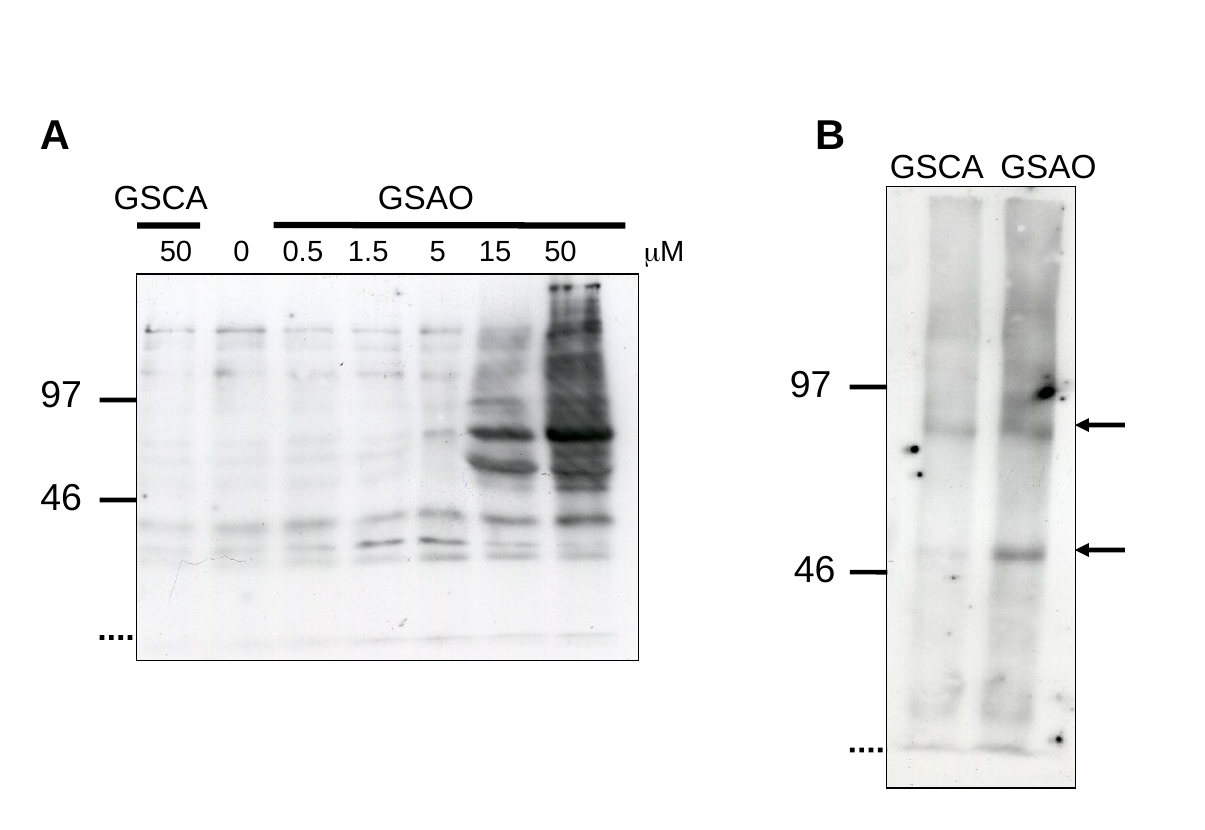

A
B
GSCA GSAO
GSCA
GSAO
 50 0 0.5 1.5 5 15 50 μM
97
97
46
46

Supplement: Additional File 6 — Detection of proteins hyperphosphorylated after treatment of PWBC with GSAO using a PKA substrate-directed antibody. Cells were incubated for 24 h with GSAO or GSCA as indicated and analysed with an antibody made to recognise multiple PKA substrate proteins in their PKA-phosphorylated forms (R-R-x-pS or R-x-x-pT motif, Cell Signalling Technology 9621). A Strong effect of GSAO on phosphoproteins detected with the PKC substrate phospho-antibody using protein extracts from low serum cultured PWBC. The control compound GSCA does not detectably change the basal phospho-protein pattern. B Effects of 15 μM GSAO on high serum cultured PWBC. Arrows indicate bands that are slightly hyperphosphorylated in GSAO-treated cells. [file 1471-2407-6-155-S6.ppt]

## Slide 1
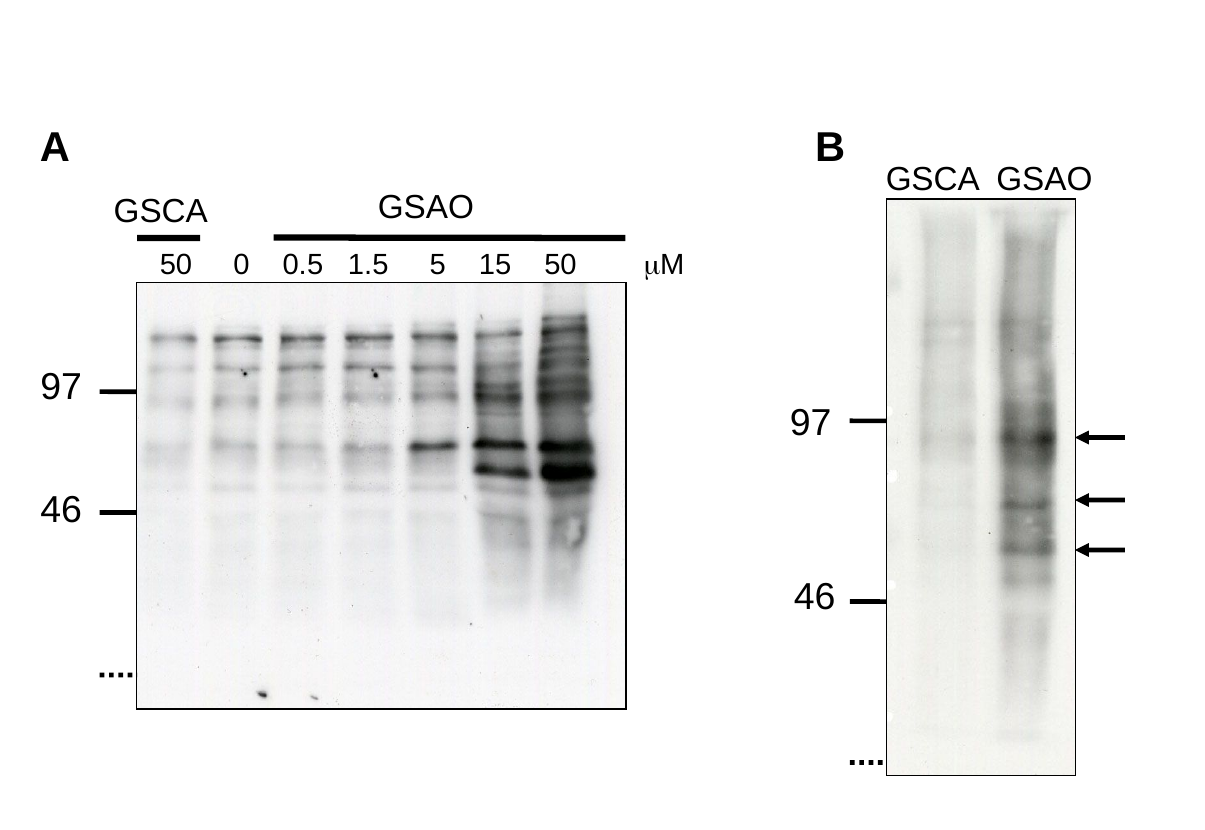

A
B
GSCA GSAO
GSAO
GSCA
 50 0 0.5 1.5 5 15 50 μM
97
97
46
46

Supplement: Additional File 7 — Detection of proteins hyper-phosphorylated after treatment of PWBC with GSAO using a PKD substrate-directed antibody. Cells were incubated for 24 h with GSAO or GSCA as indicated and analysed with an antibody made to recognise multiple PKD substrate proteins in their PKD-phosphorylated forms (L-x-R-x-x-pT/pS motif, Cell Signalling Technology 4381) A Effect of GSAO on phosphoproteins detected with the PKC substrate phospho-antibody using protein extracts from low serum cultured PWBC. The control compound GSCA does not detectably change the basal phospho-protein pattern. B Effects of 15 μM GSAO on high serum cultured PWBC. Arrows indicate three bands that are clearly hyperphosphorylated in GSAO-treated cells. [file 1471-2407-6-155-S7.ppt]
