# Supplementary material for: Molecular profiling of signalling proteins for effects induced by the anti-cancer compound GSAO with 400 antibodies
Source: BMC Cancer. 2006 Jun 9;6:155. doi: 10.1186/1471-2407-6-155 (PMC1550423; doi:10.1186/1471-2407-6-155)
Supplement: Additional File 5 — Effects of GSAO on the phosphorylation of p90Rsk at Ser380 in PWBC obtained from different volunteers. PWBC were treated with GSAO or GSCA as indicated for 24 h and lysates analysed for the phosphorylation of Ser380 in p90Rsk. Cells from three different donors were used. The male donor was analysed on two different occasions. Increased Ser380 phosphorylation is clearly visible at 5 μM GSAO in all cases. With 50 μM GSAO a major mobility shift of p90Rsk (pSer380) is visible. This could indicate the occurrence of additional phosphorylation events. [file 1471-2407-6-155-S5.ppt]

## Slide 1
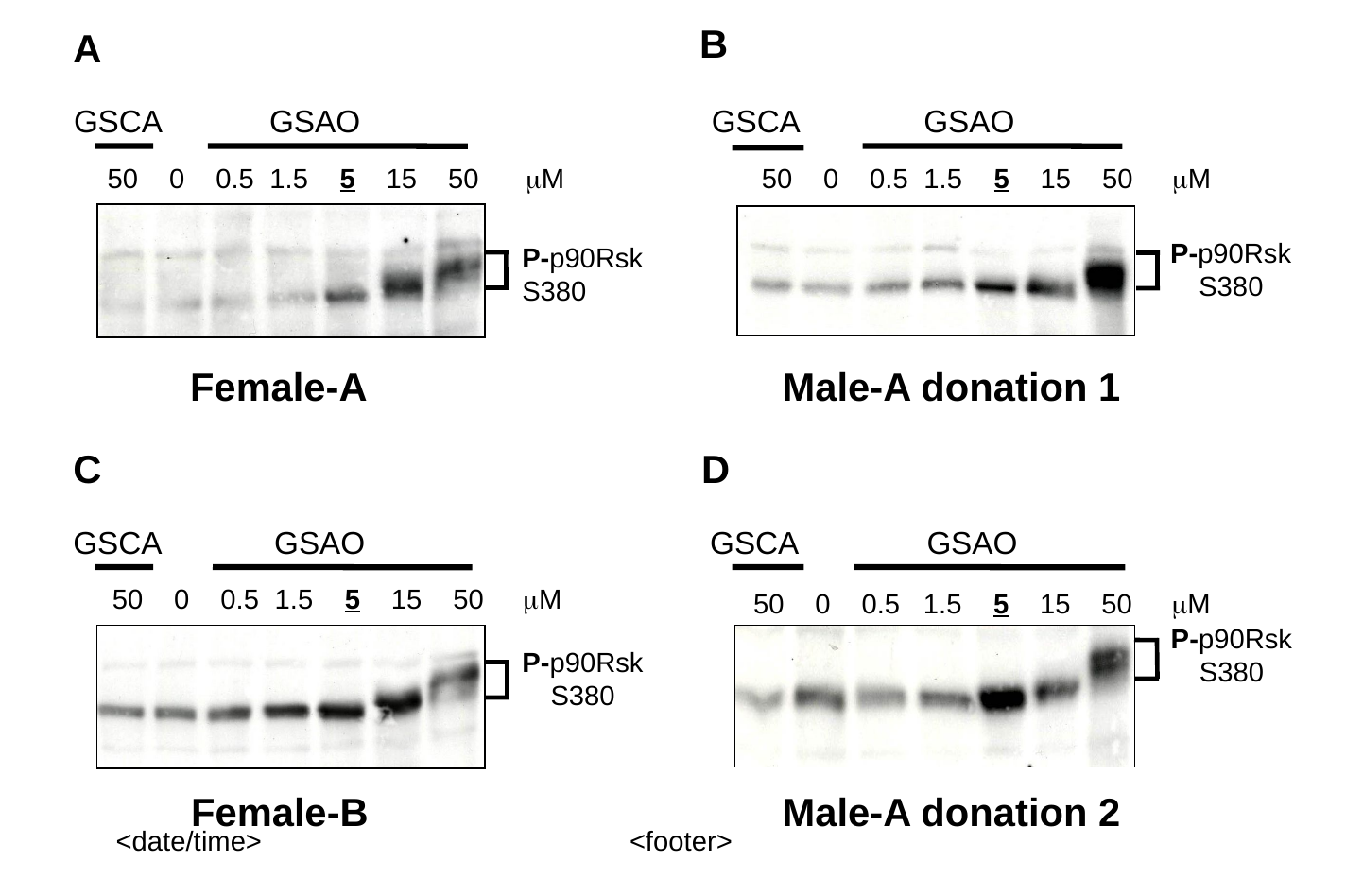

B
A
GSCA
GSAO
GSCA
GSAO
 50 0 0.5 1.5 5 15 50 μM
 50 0 0.5 1.5 5 15 50 μM
P-p90Rsk
S380
P-p90Rsk
S380
Female-A
Male-A donation 1
C
D
GSCA
GSAO
GSCA
GSAO
 50 0 0.5 1.5 5 15 50 μM
 50 0 0.5 1.5 5 15 50 μM
P-p90Rsk
S380
P-p90Rsk
S380
Female-B
Male-A donation 2
<date/time>
<footer>
