# Supplementary material for: Molecular profiling of signalling proteins for effects induced by the anti-cancer compound GSAO with 400 antibodies
Source: BMC Cancer. 2006 Jun 9;6:155. doi: 10.1186/1471-2407-6-155 (PMC1550423; doi:10.1186/1471-2407-6-155)
Supplement: Additional File 10 — Summary of GSAO-induced protein expression changes in PWBC cultured with low serum medium. Of 240 individually selected signalling proteins, which were analysed by the commercially available Powerblot™, the 24 proteins listed here showed an apparent change in protein expression considered to be highly significant according to the company. Proteins in blue color are upregulated, proteins in black downregulated with GSAO vs. GSCA. From this list it is obvious, that kinases and PTPases and their targets make up the majority of proteins affected in their expression level by GSAO. At least two of the signals (LAT, VASP) are, however, clearly questionable, as the detected band size in the sample varies greatly from the expected protein size. In other cases, for example Hic-5, an expression change is detected by the automated system. Upon visual inspection this supposed expression change results from a mobility shift of Hic 5. A full list of all proteins analysed and changes detected are compiled in additional file 8. [file 1471-2407-6-155-S10.ppt]

## Slide 1
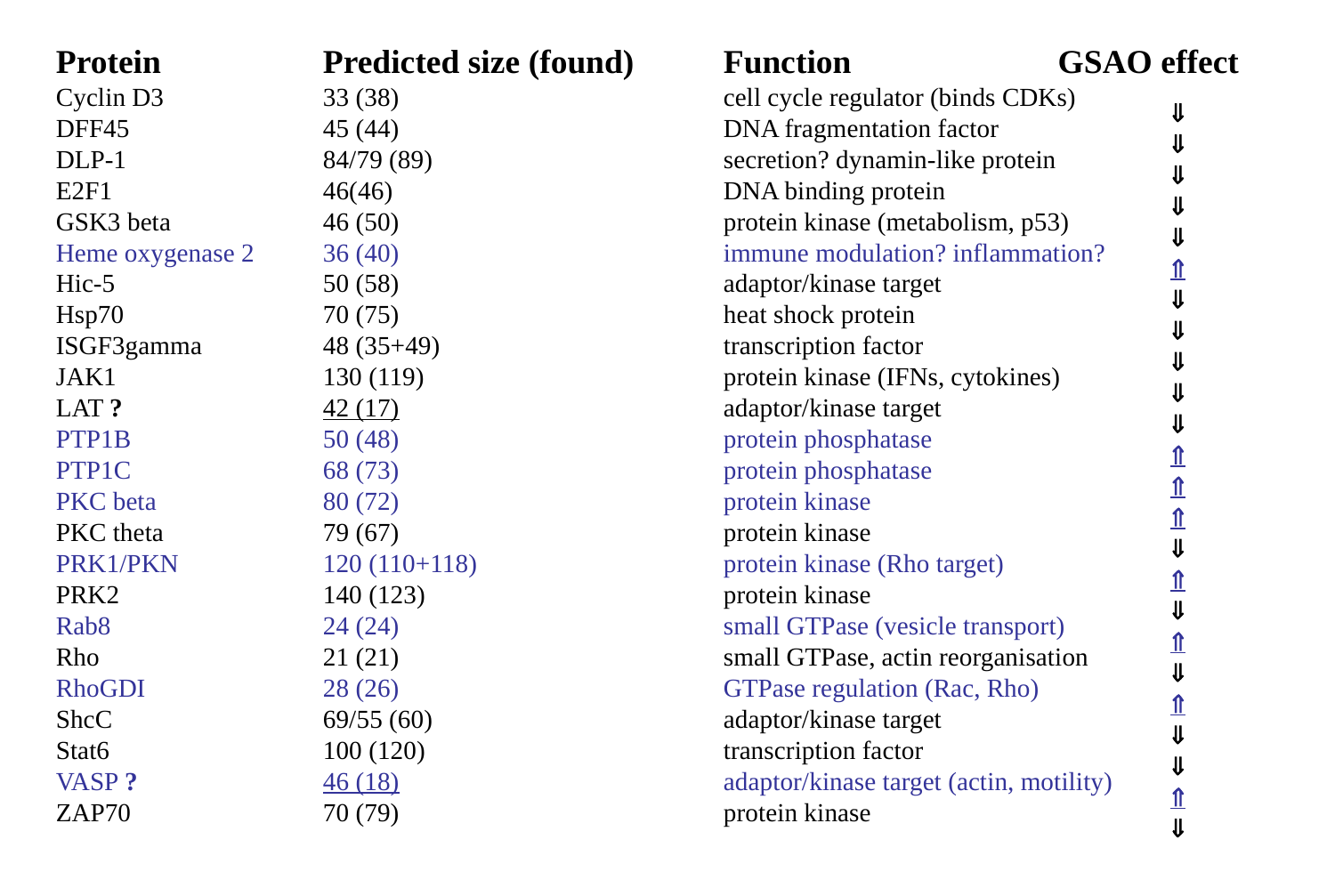

Protein		Predicted size (found)	Function		 GSAO effect
Cyclin D3		33 (38)			cell cycle regulator (binds CDKs)
DFF45		45 (44)			DNA fragmentation factor
DLP-1		84/79 (89)			secretion? dynamin-like protein
E2F1		46(46)			DNA binding protein
GSK3 beta		46 (50)			protein kinase (metabolism, p53)
Heme oxygenase 2	36 (40)			immune modulation? inflammation?
Hic-5		50 (58)			adaptor/kinase target
Hsp70		70 (75)			heat shock protein
ISGF3gamma	48 (35+49)			transcription factor
JAK1		130 (119)			protein kinase (IFNs, cytokines)
LAT ?		42 (17)			adaptor/kinase target
PTP1B		50 (48)			protein phosphatase
PTP1C		68 (73)			protein phosphatase
PKC beta		80 (72)			protein kinase
PKC theta		79 (67)			protein kinase
PRK1/PKN		120 (110+118)		protein kinase (Rho target)
PRK2		140 (123)			protein kinase
Rab8		24 (24)			small GTPase (vesicle transport)
Rho		21 (21)			small GTPase, actin reorganisation
RhoGDI		28 (26)			GTPase regulation (Rac, Rho)
ShcC		69/55 (60)			adaptor/kinase target
Stat6		100 (120)			transcription factor
VASP ?		46 (18)			adaptor/kinase target (actin, motility)
ZAP70		70 (79)			protein kinase

