# Supplementary material for: Molecular profiling of signalling proteins for effects induced by the anti-cancer compound GSAO with 400 antibodies
Source: BMC Cancer. 2006 Jun 9;6:155. doi: 10.1186/1471-2407-6-155 (PMC1550423; doi:10.1186/1471-2407-6-155)
Supplement: Additional File 11 — Pleiotropic effects on PWBC in low serum conditions. [file 1471-2407-6-155-S11.doc]

## Pleiotropic effects of GSAO on PWBC cultured in low serum conditions (additional results)

The effects of GSAO vs. GSCA on specific signalling proteins in PWBC were investigated. As the arsenite in GSAO is essential for its *in vitro* and *in vivo* activity, we focussed initially on members of the MAPK family of protein kinases, which are well known to respond to treatment of cells with PAO or arsenic trioxide [1-5], namely JNKs (a. k. a. SAPKs), p38 and Erk1/2. The JNK family kinases p46 (JNK1) and p54 (JNK2/3) were clearly phosphorylated on the regulatory epitope Thr183/Tyr185 upon treatment of PWBC with 5 μM or more of GSAO, but not by application of GSCA (additional file 3). On the other hand, p38 phosphorylation on the epitope Thr180/Tyr182 did not appear to be affected (data not shown). Phosphorylation of p44 (Erk1) and p42 (Erk2) on Thr202/Tyr204 was clearly elevated by exposure of PWBC to 15 μM GSAO or more (additional file 4A and B). Erk1/2 phosphorylation was analysed using two different phosphoepitope-specific Erk antibodies. Both reagents gave similar results, but the polyclonal antibody (additional file 4A) recognised additional bands at other molecular weights, which were affected by exposure of cells to GSAO. These bands possibly represent some other Erk kinase family members. Extra bands of unexpected size were also seen with other commercially available phospho-epitope antibodies used throughout this study (data not shown). In some cases, the most prominent changes in phosphorylation were detected for proteins of unexpected molecular weight. These findings highlight the importance of size separation for the phosphoproteins to be analysed. Array- or FACS-type assays would possibly fail to give reliable data with the antibodies currently available in a very significant number of cases.

Since Erk1/2 are considered to be intimately linked functionally with their upstream MEK kinases, MEK1/2 phosphorylation after exposure of PWBC to GSAO or GSCA was also analysed. Unexpectedly, we failed to see clear changes in the phosphorylation of the activity-regulating epitope S217/S221 in MEK1/2, although the antibody was clearly functional in other experiments (data not shown). This result could indicate that another kinase is involved in Erk1/2 activation by GSAO. Alternatively, GSAO could inhibit phosphatases which regulate Erk1/2, for example members of the MKP family [6]. A variety of more indirect regulatory mechanisms may also explain the apparent MEK-independence of Erk activation induced by GSAO.

c-Raf-1, an important upstream kinase of MEK1/2, was also analysed with two commercially available phospho-specific antibodies. Dephosphorylation of Ser259 is considered to be important for activation of c-Raf-1, while Tyr340/Tyr341 phosphorylation contributes to its activation. As shown in additional file 4C, phosphorylation of the epitope Tyr340/Tyr341 is induced by GSAO. On the other hand, dephosphorylation of the pSer259-epitope was not readily observable (additional file 4D). Complicating the matter, a change in Raf protein mobility is apparent upon GSAO treatment of PWBC. This could prevent the detection of moderate changes in Raf Ser259 phosphorylation.

The Erk1/2 kinases are known to have a wide variety of substrate proteins in cells [7-11]. Several of these target proteins were analysed with phospho-epitope specific antibodies. One example, phosphorylation of the Ser/Thr kinase p90Rsk on Ser380, is shown in additional file 5. Results obtained with PWBC from 3 different volunteers are shown. The results were quite similar in all cases. Increased phosphorylation of Ser380 on p90Rsk was clearly detectable at 5 μM GSAO. At higher compound concentrations a p90Rsk mobility shift is obvious, possibly the result of additional phosphorylation events. Other known Erk1/2 targets were also affected by GSAO (results included in additional file 9).

GSAO may not only modify the level of phosphorylation in some signalling proteins, but could also affect protein expression. This was analysed by blotting GSAO-treated PWBC lysates with a panel of 240 selected commercial monoclonal antibodies (BD PowerBlot). Twenty-four of 240 antibodies showed a change that was considered to be significant by the PowerBlot software (level 8 to 10; full details in additional file 8), although some immunoreactive bands appeared at unexpected molecular weights. The 24 proteins detected by blotting with the corresponding mAbs are listed in additional file 10. Visual inspection of the results showed that the apparent changes in abundance detected by the commercial software of the PowerBlot system often appears to result from changes in protein mobility (possibly reflecting phosphorylation events) rather than from actual changes in protein abundance. From these data, combined with further results discussed in more detail below, we conclude that true changes in the abundance of signalling molecules are likely to account for only a small fraction of the GSAO effects observed. Thus, a broad analysis of activity changes in signalling proteins upon GSAO treatment appears to be a more promising approach to elucidate the molecular effects of this compound. Activity changes of signalling proteins can often be rapidly monitored using antibodies, which recognise specific protein modifications, proteolytic cleavage sites, or specific protein conformations.

In subsequent experiments, a large panel of phospho-epitope specific antibodies was used to further profile the molecular actions of GSAO. The results are summarised in additional file 9. From these data it became apparent that a large percentage of the proteins analysed is affected by GSAO, often at concentrations as low as 5 μM. A subpanel of antibodies was further analysed for inter-volunteer variation of the GSAO response and some individual differences were found, suggesting that GSAO may exert variable effects on cell signalling of different individuals (data not shown). The control compound GSCA applied at 50 μM did not affect any of the analysed protein phosphorylations detectably.

# References

1. Bernstam L, Nriagu J: **Molecular aspects of arsenic stress**. *J Toxicol Environ Health B Crit Rev* 2000, **3**(4):293-322.

2. Cavigelli M, Li WW, Lin A, Su B, Yoshioka K, Karin M: **The tumor promoter arsenite stimulates AP-1 activity by inhibiting a JNK phosphatase**. *Embo J* 1996, **15**(22):6269-6279.

3. Ludwig S, Hoffmeyer A, Goebeler M, Kilian K, Hafner H, Neufeld B, Han J, Rapp UR: **The stress inducer arsenite activates mitogen-activated protein kinases extracellular signal-regulated kinases 1 and 2 via a MAPK kinase 6/p38-dependent pathway**. *J Biol Chem* 1998, **273**(4):1917-1922.

4. Porter AC, Fanger GR, Vaillancourt RR: **Signal transduction pathways regulated by arsenate and arsenite**. *Oncogene* 1999, **18**(54):7794-7802.

5. Rouse J, Cohen P, Trigon S, Morange M, Alonso-Llamazares A, Zamanillo D, Hunt T, Nebreda AR: **A novel kinase cascade triggered by stress and heat shock that stimulates MAPKAP kinase-2 and phosphorylation of the small heat shock proteins**. *Cell* 1994, **78**(6):1027-1037.

6. Farooq A, Zhou MM: **Structure and regulation of MAPK phosphatases**. *Cell Signal* 2004, **16**(7):769-779.

7. Dougherty MK, Muller J, Ritt DA, Zhou M, Zhou XZ, Copeland TD, Conrads TP, Veenstra TD, Lu KP, Morrison DK: **Regulation of Raf-1 by direct feedback phosphorylation**. *Mol Cell* 2005, **17**(2):215-224.

8. Hazzalin CA, Mahadevan LC: **MAPK-regulated transcription: a continuously variable gene switch?** *Nat Rev Mol Cell Biol* 2002, **3**(1):30-40.

9. Pearson G, Robinson F, Beers Gibson T, Xu BE, Karandikar M, Berman K, Cobb MH: **Mitogen-activated protein (MAP) kinase pathways: regulation and physiological functions**. *Endocr Rev* 2001, **22**(2):153-183.

10. Roux PP, Blenis J: **ERK and p38 MAPK-activated protein kinases: a family of protein kinases with diverse biological functions**. *Microbiol Mol Biol Rev* 2004, **68**(2):320-344.

11. Whitmarsh AJ, Davis RJ: **A central control for cell growth**. *Nature* 2000, **403**(6767):255-256.
